# (*S*)-[5-Methyl-3-(3-methyl­thio­phen-2-yl)-4,5-dihydro­isoxazol-5-yl]methanol

**DOI:** 10.1107/S1600536811011639

**Published:** 2011-04-07

**Authors:** Young Kwan Ko, Jae Wook Ryu, Dong Wan Koo, Jae Choon Woo, Chong-Hyeak Kim

**Affiliations:** a Biomaterials Research Center, Korea Research Institute of Chemical Technology, PO Box 107, Yuseong, Daejeon 305-600, Republic of Korea; b Drug Discovery Platform Technology Team, Korea Research Institute of Chemical Technology, PO Box 107, Yuseong, Daejeon 305-600, Republic of Korea; c Center for Chemical Analysis, Korea Research Institute of Chemical Technology, PO Box 107, Yuseong, Daejeon 305-600, Republic of Korea

## Abstract

In the title compound, C_10_H_13_NO_2_S, the thio­phene and isoxazoline rings are almost coplanar, the dihedral angle between their least-squares planes being 2.08 (1)°. The O—H atoms of the methyl hy­droxy group and the N atom of the isoxazole ring are orientated in the same direction to allow for the formation of inter­molecular O—H⋯N hydrogen bonds that lead to a supra­molecular chain along the *a* axis.

## Related literature

For the synthesis, biological activity and mode of action of herbicides, see; Ryu *et al.* (2005 ▶); Hwang *et al.* (2005 ▶); Koo *et al.* (2007 ▶); Koo & Hwang (2008 ▶). For relevant reviews of herbicides, see; Boger *et al.* (2002 ▶); Bryant & Bite (2010 ▶).
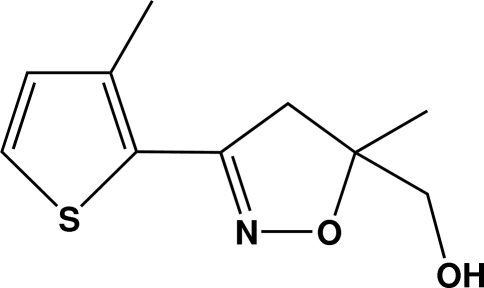


## Experimental

### 

#### Crystal data

C_10_H_13_NO_2_S
*M* *_r_* = 211.27
Orthorhombic, 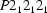

*a* = 7.3672 (9) Å
*b* = 8.8534 (11) Å
*c* = 16.0632 (19) Å
*V* = 1047.7 (2) Å^3^
*Z* = 4
Mo *K*α radiation
μ = 0.28 mm^−1^
*T* = 296 K
0.39 × 0.20 × 0.11 mm

#### Data collection

Bruker APEXII CCD diffractometer
Absorption correction: multi-scan (*SADABS*; Sheldrick, 1996 ▶) *T*_min_ = 0.898, *T*_max_ = 0.970
11038 measured reflections
2619 independent reflections
2096 reflections with *I* > 2σ(*I*)
*R*_int_ = 0.025

#### Refinement

*R*[*F* ^2^ > 2σ(*F* ^2^)] = 0.047
*wR*(*F* ^2^) = 0.149
*S* = 1.08
2619 reflections
127 parameters
H-atom parameters constrained
Δρ_max_ = 0.47 e Å^−3^
Δρ_min_ = −0.36 e Å^−3^
Absolute structure: Flack (1983 ▶), 1087 Friedel pairs
Flack parameter: 0.02 (14)

### 

Data collection: *APEX2* (Bruker, 2009 ▶); cell refinement: *SAINT* (Bruker, 2009 ▶); data reduction: *SAINT*; program(s) used to solve structure: *SHELXS97* (Sheldrick, 2008 ▶); program(s) used to refine structure: *SHELXL97* (Sheldrick, 2008 ▶); molecular graphics: *XP* in *SHELXTL* (Sheldrick, 2008 ▶); software used to prepare material for publication: *SHELXL97*.

## Supplementary Material

Crystal structure: contains datablocks global, I. DOI: 10.1107/S1600536811011639/tk2733sup1.cif

Structure factors: contains datablocks I. DOI: 10.1107/S1600536811011639/tk2733Isup2.hkl

Additional supplementary materials:  crystallographic information; 3D view; checkCIF report

## Figures and Tables

**Table 1:** Hydrogen-bond geometry (Å, °)

*D*—H⋯*A*	*D*—H	H⋯*A*	*D*⋯*A*	*D*—H⋯*A*
O13—H13⋯N7^i^	0.82	2.17	2.905 (3)	150

Symmetry code: (i) 
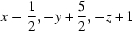
.

**Fig. 1.**
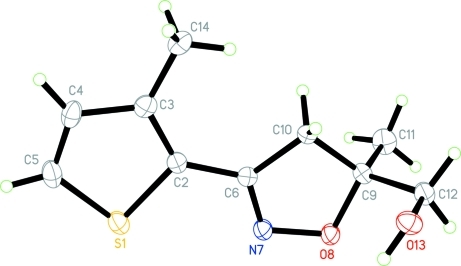
The molecular structure of the title compound with the atom numbering scheme. Displacement ellipsoids are drawn at the 30% probability level. H atoms are presented as a small spheres of arbitrary radius.

**Fig. 2.**
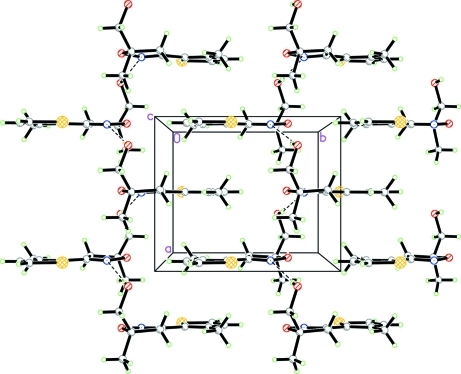
The molecular packing structure of the title compound, viewed down the c axis showing the O—H···N hydrogen bonds as dashed lines.

## supplementary crystallographic information

### Comment

Weed control is very important for the improvment of agricultural efficiency Boger *et al.*, 2002; Bryant *et al.*, 2010). A number of herbicides have been used for the purpose of weed killing. Recently a new isoxazoline herbicide MRC-01 has been developed (Ryu *et al.*, 2005; Hwang *et al.*, 2005; Koo *et al.*, 2007; Koo & Hwang, 2008; Bryant & Bite, 2010). MRC-01 was synthesized by the reaction of [5-methyl-3-(3-methylthiophen -2-yl)-4,5-dihydroisoxazol-5-yl]methanol and 2,6-difluorobenzylbromide in the presence of base. The key intermediate [5-methyl-3-(3-methylthiophen-2-yl)-4,5-dihydroisoxazol-5-yl]methanol was used as racemic compound but could be separated into enantiomers by employing chiral HPLC column technology. Herein, we report the crystal structure of title compound (Fig. 1). The thiophene ring and the isoxazole ring are almost coplanar with the dihedral angle being 2.08 (1) °. The conformation of the O—H of the methyl hydroxy group and the N atom of the isoxazole ring are in the same direction to allow intermolecular hydrogen bonds to form. In the crystal structure (Fig. 2), the molecules are linked by these O—H···N hydrogen bonds into a one-dimensional chain running along the *a* axis.

### Experimental

The title compound was obtained by a chiral separation of racemic [5-methyl-3-(3-methylthiophen-2-yl)-4,5-dihydroisoxazol-5-yl]methanol employing chiral prep-HPLC under the condition shown below. HPLC conditions: Column: (*R*,*R*) WHELK-01 (25 cm *x* 10.0 mm). Regis.Co.; Eluent: 25% 2-propanol + 75% n-hexane; Flow Rate 4.0 ml/min; Detection: 254 nm; Injection volume: 0.1 ml. The first eluting fraction was concentrated under reduced pressure to provide the title compound [α]_D_ -59.95 (c = 1, dichloromethane). Single crystals suitable for X-ray diffraction were prepared by recrystallization from its ethyl acetate solution at room temperature.

### Refinement

All hydrogen atoms were placed in calculated positions using a riding model, with C—H = 0.93–0.97 Å and O—H = 0.82 Å, and with *U*_iso_(H) = 1.2–1.5 *U*_eq_(C, O).

### Crystal data

**Table:**

C_10_H_13_NO_2_S	*F*(000) = 448
*M**_r_* = 211.27	*D*_x_ = 1.339 Mg m^−^^3^
Orthorhombic, *P*2_1_2_1_2_1_	Mo *K*α radiation, λ = 0.71073 Å
Hall symbol: P 2ac 2ab	Cell parameters from 4205 reflections
*a* = 7.3672 (9) Å	θ = 2.5–26.1°
*b* = 8.8534 (11) Å	µ = 0.28 mm^−^^1^
*c* = 16.0632 (19) Å	*T* = 296 K
*V* = 1047.7 (2) Å^3^	Block, silver
*Z* = 4	0.39 × 0.20 × 0.11 mm

### Data collection

**Table:**

Bruker APEXII CCD diffractometer	2619 independent reflections
Radiation source: fine-focus sealed tube	2096 reflections with *I* > 2σ(*I*)
graphite	*R*_int_ = 0.025
φ and ω scans	θ_max_ = 28.4°, θ_min_ = 2.5°
Absorption correction: multi-scan (*SADABS*; Sheldrick, 1996)	*h* = −6→9
*T*_min_ = 0.898, *T*_max_ = 0.970	*k* = −11→11
11038 measured reflections	*l* = −21→21

### Refinement

**Table:**

Refinement on *F*^2^	Secondary atom site location: difference Fourier map
Least-squares matrix: full	Hydrogen site location: inferred from neighbouring sites
*R*[*F*^2^ > 2σ(*F*^2^)] = 0.047	H-atom parameters constrained
*wR*(*F*^2^) = 0.149	*w* = 1/[σ^2^(*F*_o_^2^) + (0.086*P*)^2^ + 0.1644*P*] where *P* = (*F*_o_^2^ + 2*F*_c_^2^)/3
*S* = 1.08	(Δ/σ)_max_ < 0.001
2619 reflections	Δρ_max_ = 0.47 e Å^−^^3^
127 parameters	Δρ_min_ = −0.36 e Å^−^^3^
0 restraints	Absolute structure: Flack (1983), 1087 Friedel pairs
Primary atom site location: structure-invariant direct methods	Flack parameter: 0.02 (14)

### Special details

**Table:**

Geometry. All e.s.d.'s (except the e.s.d. in the dihedral angle between two l.s. planes) are estimated using the full covariance matrix. The cell e.s.d.'s are taken into account individually in the estimation of e.s.d.'s in distances, angles and torsion angles; correlations between e.s.d.'s in cell parameters are only used when they are defined by crystal symmetry. An approximate (isotropic) treatment of cell e.s.d.'s is used for estimating e.s.d.'s involving l.s. planes.
Refinement. Refinement of *F*^2^ against ALL reflections. The weighted *R*-factor *wR* and goodness of fit *S* are based on *F*^2^, conventional *R*-factors *R* are based on *F*, with *F* set to zero for negative *F*^2^. The threshold expression of *F*^2^ > σ(*F*^2^) is used only for calculating *R*-factors(gt) *etc*. and is not relevant to the choice of reflections for refinement. *R*-factors based on *F*^2^ are statistically about twice as large as those based on *F*, and *R*- factors based on ALL data will be even larger.

### Fractional atomic coordinates and isotropic or equivalent isotropic displacement parameters (Å^2^)

**Table:**

	*x*	*y*	*z*	*U*_iso_*/*U*_eq_	
S1	−0.00757 (12)	0.90745 (7)	0.64150 (4)	0.0503 (2)	
C2	−0.0074 (4)	0.8815 (3)	0.53306 (13)	0.0390 (4)	
C3	−0.0070 (5)	0.7321 (3)	0.51314 (15)	0.0446 (5)	
C4	−0.0075 (4)	0.6395 (3)	0.58618 (17)	0.0525 (6)	
H4A	−0.0080	0.5345	0.5841	0.063*	
C5	−0.0071 (5)	0.7169 (3)	0.65749 (17)	0.0568 (6)	
H5	−0.0067	0.6721	0.7099	0.068*	
C6	−0.0104 (4)	1.0139 (2)	0.47984 (12)	0.0368 (4)	
N7	−0.0045 (4)	1.1462 (2)	0.51234 (11)	0.0448 (4)	
O8	−0.0178 (4)	1.25711 (18)	0.45051 (10)	0.0490 (5)	
C9	−0.0104 (4)	1.1857 (3)	0.36792 (13)	0.0405 (5)	
C10	−0.0244 (4)	1.0174 (3)	0.38670 (13)	0.0423 (6)	
H10A	−0.1394	0.9763	0.3679	0.051*	
H10B	0.0740	0.9615	0.3610	0.051*	
C11	0.1691 (4)	1.2299 (4)	0.3280 (2)	0.0630 (9)	
H11A	0.1707	1.3369	0.3182	0.094*	
H11B	0.1828	1.1774	0.2760	0.094*	
H11C	0.2672	1.2033	0.3645	0.094*	
C12	−0.1672 (4)	1.2500 (4)	0.31872 (17)	0.0452 (6)	
H12A	−0.1627	1.3593	0.3219	0.054*	
H12B	−0.1527	1.2219	0.2607	0.054*	
O13	−0.3406 (2)	1.2007 (2)	0.34646 (11)	0.0489 (5)	
H13	−0.3554	1.2261	0.3951	0.073*	
C14	−0.0073 (5)	0.6707 (3)	0.42966 (17)	0.0507 (6)	
H14A	−0.0080	0.7518	0.3900	0.076*	
H14B	−0.1134	0.6093	0.4220	0.076*	
H14C	0.0994	0.6101	0.4216	0.076*	

### Atomic displacement parameters (Å^2^)

**Table:**

	*U*^11^	*U*^22^	*U*^33^	*U*^12^	*U*^13^	*U*^23^
S1	0.0500 (4)	0.0629 (4)	0.0381 (3)	0.0022 (4)	−0.0002 (4)	0.0035 (2)
C2	0.0289 (10)	0.0484 (11)	0.0399 (9)	0.0021 (13)	−0.0002 (12)	0.0035 (8)
C3	0.0314 (11)	0.0483 (12)	0.0540 (13)	0.0039 (14)	0.0010 (14)	0.0021 (9)
C4	0.0406 (12)	0.0495 (13)	0.0673 (16)	0.0052 (15)	−0.0003 (17)	0.0198 (11)
C5	0.0458 (13)	0.0715 (17)	0.0529 (13)	0.0036 (18)	0.0057 (16)	0.0222 (12)
C6	0.0320 (10)	0.0401 (10)	0.0383 (10)	−0.0011 (13)	−0.0029 (12)	−0.0017 (8)
N7	0.0555 (12)	0.0430 (9)	0.0361 (8)	0.0010 (14)	−0.0045 (13)	0.0004 (7)
O8	0.0711 (14)	0.0379 (8)	0.0380 (8)	0.0026 (11)	−0.0086 (11)	−0.0009 (6)
C9	0.0412 (11)	0.0465 (11)	0.0340 (9)	−0.0041 (14)	−0.0004 (13)	0.0005 (8)
C10	0.0525 (15)	0.0389 (10)	0.0355 (9)	0.0021 (12)	−0.0024 (12)	−0.0035 (8)
C11	0.0416 (15)	0.079 (2)	0.069 (2)	−0.0069 (16)	0.0063 (15)	0.0075 (18)
C12	0.0408 (14)	0.0556 (15)	0.0391 (12)	0.0012 (13)	0.0003 (12)	0.0045 (11)
O13	0.0397 (9)	0.0606 (11)	0.0462 (10)	−0.0008 (9)	0.0025 (8)	−0.0063 (9)
C14	0.0455 (12)	0.0429 (12)	0.0637 (14)	0.0026 (15)	0.0004 (16)	0.0016 (10)

### Geometric parameters (Å, °)

**Table:**

S1—C5	1.706 (3)	C9—C11	1.522 (4)
S1—C2	1.757 (2)	C9—C10	1.524 (3)
C2—C3	1.361 (3)	C10—H10A	0.9700
C2—C6	1.451 (3)	C10—H10B	0.9700
C3—C4	1.431 (3)	C11—H11A	0.9600
C3—C14	1.447 (3)	C11—H11B	0.9600
C4—C5	1.335 (4)	C11—H11C	0.9600
C4—H4A	0.9300	C12—O13	1.422 (3)
C5—H5	0.9300	C12—H12A	0.9700
C6—N7	1.283 (3)	C12—H12B	0.9700
C6—C10	1.500 (3)	O13—H13	0.8200
N7—O8	1.400 (2)	C14—H14A	0.9600
O8—C9	1.470 (3)	C14—H14B	0.9600
C9—C12	1.511 (4)	C14—H14C	0.9600
			
C5—S1—C2	91.14 (12)	C6—C10—H10A	111.3
C3—C2—C6	130.3 (2)	C9—C10—H10A	111.3
C3—C2—S1	111.12 (17)	C6—C10—H10B	111.3
C6—C2—S1	118.57 (17)	C9—C10—H10B	111.3
C2—C3—C4	111.4 (2)	H10A—C10—H10B	109.2
C2—C3—C14	125.7 (2)	C9—C11—H11A	109.5
C4—C3—C14	123.0 (2)	C9—C11—H11B	109.5
C5—C4—C3	114.1 (2)	H11A—C11—H11B	109.5
C5—C4—H4A	122.9	C9—C11—H11C	109.5
C3—C4—H4A	122.9	H11A—C11—H11C	109.5
C4—C5—S1	112.24 (19)	H11B—C11—H11C	109.5
C4—C5—H5	123.9	O13—C12—C9	114.0 (2)
S1—C5—H5	123.9	O13—C12—H12A	108.7
N7—C6—C2	119.83 (19)	C9—C12—H12A	108.7
N7—C6—C10	112.94 (18)	O13—C12—H12B	108.7
C2—C6—C10	127.22 (19)	C9—C12—H12B	108.7
C6—N7—O8	110.42 (17)	H12A—C12—H12B	107.6
N7—O8—C9	109.65 (16)	C12—O13—H13	109.5
O8—C9—C12	106.4 (2)	C3—C14—H14A	109.5
O8—C9—C11	107.6 (2)	C3—C14—H14B	109.5
C12—C9—C11	110.33 (19)	H14A—C14—H14B	109.5
O8—C9—C10	103.84 (16)	C3—C14—H14C	109.5
C12—C9—C10	114.8 (2)	H14A—C14—H14C	109.5
C11—C9—C10	113.2 (3)	H14B—C14—H14C	109.5
C6—C10—C9	102.27 (17)		
			
C5—S1—C2—C3	0.0 (3)	C2—C6—N7—O8	−177.1 (3)
C5—S1—C2—C6	179.1 (2)	C10—C6—N7—O8	1.6 (4)
C6—C2—C3—C4	−178.8 (3)	C6—N7—O8—C9	−7.3 (3)
S1—C2—C3—C4	0.2 (4)	N7—O8—C9—C12	131.1 (2)
C6—C2—C3—C14	0.8 (6)	N7—O8—C9—C11	−110.7 (3)
S1—C2—C3—C14	179.8 (3)	N7—O8—C9—C10	9.5 (3)
C2—C3—C4—C5	−0.4 (4)	N7—C6—C10—C9	4.4 (4)
C14—C3—C4—C5	−180.0 (3)	C2—C6—C10—C9	−177.1 (3)
C3—C4—C5—S1	0.4 (4)	O8—C9—C10—C6	−8.0 (3)
C2—S1—C5—C4	−0.2 (3)	C12—C9—C10—C6	−123.7 (2)
C3—C2—C6—N7	−177.8 (4)	C11—C9—C10—C6	108.3 (3)
S1—C2—C6—N7	3.3 (4)	O8—C9—C12—O13	−70.1 (3)
C3—C2—C6—C10	3.7 (5)	C11—C9—C12—O13	173.5 (3)
S1—C2—C6—C10	−175.2 (3)	C10—C9—C12—O13	44.2 (3)

### Hydrogen-bond geometry (Å, °)

**Table:**

*D*—H···*A*	*D*—H	H···*A*	*D*···*A*	*D*—H···*A*
O13—H13···N7^i^	0.82	2.17	2.905 (3)	150

Symmetry codes: (i) *x*−1/2, −*y*+5/2, −*z*+1.
